# Supplementary material for: Discovery and application of insertion-deletion (INDEL) polymorphisms for QTL mapping of early life-history traits in Atlantic salmon
Source: BMC Genomics. 2010 Mar 8;11:156. doi: 10.1186/1471-2164-11-156 (PMC2838853; doi:10.1186/1471-2164-11-156)
Supplement: Additional file 2 — Information on developed 76 locus single-run INDEL panel in Atlantic salmon. Information on fluorescence labeling, primer concentrations, PCR pooling and links to alignments, INDEL motifs and GENESCAN (Burge and Karlin 1997) predictions of genes/exons are available in html format. [file 1471-2164-11-156-S2.ZIP › Additionalfile2/snpsummary3019.html]

```
Cluster 606 Contig 1

prev  Summary    Contig List  next
```

Size of Consensus sequence = 2093

Number of sequences = 37

Minimum redundancy = 6

Key

A gi|54021288|gb|CV429079.1|CV429079 EST02308 Atlantic salmon Lambda Zap Express heart cDNA library Salmo salar cDNA clone hr\_006\_f02 5', mRNA sequence  
B gi|117517493|gb|EG849252.1|EG849252 EST\_ssal\_eve\_48156 ssaleve thyroid Salmo salar cDNA Salmo salar cDNA clone ssal\_eve\_565\_205\_rev 5', mRNA sequence  
C gi|117865923|gb|EG938619.1|EG938619 EST\_ssal\_evf\_22325 ssalevf mixed\_tissue Salmo salar cDNA Salmo salar cDNA clone ssal\_evf\_529\_149\_rev 5', mRNA sequence  
D gi|24354088|gb|CA047918.1|CA047918 ssalpitl503098 head Salmo salar cDNA, mRNA sequence  
E gi|117539556|gb|EG871001.1|EG871001 EST\_ssal\_eve\_32093 ssaleve thyroid Salmo salar cDNA Salmo salar cDNA clone ssal\_eve\_543\_216\_fwd 3', mRNA sequence  
F gi|117539557|gb|EG871002.1|EG871002 EST\_ssal\_eve\_32094 ssaleve thyroid Salmo salar cDNA Salmo salar cDNA clone ssal\_eve\_543\_216\_rev 5', mRNA sequence  
G gi|85042868|gb|DW571046.1|DW571046 EST\_ssal\_rgb2\_35465 rgb2 Salmo salar cDNA clone ssal\_rgb2\_557\_130\_fwd 3', mRNA sequence  
H gi|117462236|gb|EG794455.1|EG794455 EST\_ssal\_evd\_53936 ssalevd thymus Salmo salar cDNA Salmo salar cDNA clone ssal\_evd\_572\_265\_rev 5', mRNA sequence  
I gi|117462225|gb|EG794444.1|EG794444 EST\_ssal\_evd\_53935 ssalevd thymus Salmo salar cDNA Salmo salar cDNA clone ssal\_evd\_572\_265\_fwd 3', mRNA sequence  
J gi|29313569|gb|CB502343.1|CB502343 ssalmge501169 gut Salmo salar cDNA, mRNA sequence  
K gi|117449800|gb|EG782019.1|EG782019 EST\_ssal\_evd\_38601 ssalevd thymus Salmo salar cDNA Salmo salar cDNA clone ssal\_evd\_551\_216\_rev 5', mRNA sequence  
L gi|117510088|gb|EG841847.1|EG841847 EST\_ssal\_eve\_4556 ssaleve thyroid Salmo salar cDNA Salmo salar cDNA clone ssal\_eve\_505\_078\_rev 5', mRNA sequence  
M gi|117566102|gb|EG887078.1|EG887078 EST\_ssal\_evf\_45271 ssalevf mixed\_tissue Salmo salar cDNA Salmo salar cDNA clone ssal\_evf\_560\_195\_rev 5', mRNA sequence  
N gi|29327939|gb|CB516713.1|CB516713 ssalrgb555080 mixed\_tissue Salmo salar cDNA, mRNA sequence  
O gi|117431778|gb|EG764002.1|EG764002 EST\_ssal\_sjb\_1922 ssalsjb mixed\_tissue Salmo salar cDNA Salmo salar cDNA clone ssal\_sjb\_007\_271\_rev 5', mRNA sequence  
P gi|117828756|gb|EG901452.1|EG901452 EST\_ssal\_evf\_40208 ssalevf mixed\_tissue Salmo salar cDNA Salmo salar cDNA clone ssal\_evf\_553\_261\_rev 5', mRNA sequence  
Q gi|117502235|gb|EG834031.1|EG834031 EST\_ssal\_eve\_10811 ssaleve thyroid Salmo salar cDNA Salmo salar cDNA clone ssal\_eve\_514\_257\_rev 5', mRNA sequence  
R gi|117534547|gb|EG865992.1|EG865992 EST\_ssal\_eve\_27585 ssaleve thyroid Salmo salar cDNA Salmo salar cDNA clone ssal\_eve\_537\_190\_rev 5', mRNA sequence  
S gi|117850820|gb|EG923516.1|EG923516 EST\_ssal\_evf\_6321 ssalevf mixed\_tissue Salmo salar cDNA Salmo salar cDNA clone ssal\_evf\_506\_301\_rev 5', mRNA sequence  
T gi|29326480|gb|CB515254.1|CB515254 ssalrgb553185 mixed\_tissue Salmo salar cDNA, mRNA sequence  
U gi|84567826|gb|DW339445.1|DW339445 SGP300690 Atlantic salmon Ovaries cDNA library Salmo salar cDNA clone KG4-2446 5', mRNA sequence  
V gi|117850819|gb|EG923515.1|EG923515 EST\_ssal\_evf\_6320 ssalevf mixed\_tissue Salmo salar cDNA Salmo salar cDNA clone ssal\_evf\_506\_301\_fwd 3', mRNA sequence  
W gi|117517382|gb|EG849141.1|EG849141 EST\_ssal\_eve\_48155 ssaleve thyroid Salmo salar cDNA Salmo salar cDNA clone ssal\_eve\_565\_205\_fwd 3', mRNA sequence  
X gi|117510089|gb|EG841848.1|EG841848 EST\_ssal\_eve\_4557 ssaleve thyroid Salmo salar cDNA Salmo salar cDNA clone ssal\_eve\_505\_078\_fwd 3', mRNA sequence  
Y gi|45320378|gb|CK890645.1|CK890645 SGP150881 Atlantic salmon Ovaries cDNA library Salmo salar cDNA clone KG5-0889 5', mRNA sequence  
Z gi|117449801|gb|EG782020.1|EG782020 EST\_ssal\_evd\_38602 ssalevd thymus Salmo salar cDNA Salmo salar cDNA clone ssal\_evd\_551\_216\_fwd 3', mRNA sequence  
a gi|15843858|gb|BG934030.1|BG934030 SK1-0314 Atlantic Salmon kidney Salmo salar cDNA clone SK1-0314 similar to Endoplasmic reticulum glycoprotein, mRNA sequence  
b gi|84985327|gb|DW535677.1|DW535677 EST\_ssal\_rgb2\_96 rgb2 Salmo salar cDNA clone ssal\_rgb2\_501\_066\_rev 5', mRNA sequence  
c gi|84985328|gb|DW535678.1|DW535678 EST\_ssal\_rgb2\_97 rgb2 Salmo salar cDNA clone ssal\_rgb2\_501\_067\_rev 5', mRNA sequence  
d gi|117502223|gb|EG834019.1|EG834019 EST\_ssal\_eve\_10810 ssaleve thyroid Salmo salar cDNA Salmo salar cDNA clone ssal\_eve\_514\_257\_fwd 3', mRNA sequence  
e gi|85042867|gb|DW571045.1|DW571045 EST\_ssal\_rgb2\_35464 rgb2 Salmo salar cDNA clone ssal\_rgb2\_557\_130\_rev 5', mRNA sequence  
f gi|89862120|gb|DY718243.1|DY718243 EST\_ssal\_rgb2\_73982 ssalrgb2 mixed\_tissue Salmo salar cDNA Salmo salar cDNA clone ssal\_rgb2\_620\_213\_rev 5', mRNA sequence  
g gi|117534548|gb|EG865993.1|EG865993 EST\_ssal\_eve\_27586 ssaleve thyroid Salmo salar cDNA Salmo salar cDNA clone ssal\_eve\_537\_190\_fwd 3', mRNA sequence  
h gi|57126585|gb|CX358026.1|CX358026 ssalrgb553185\_rev\_0 mixed\_tissue Salmo salar cDNA, mRNA sequence  
i gi|15844426|gb|BG934598.1|BG934598 SK1-0910 Atlantic Salmon kidney Salmo salar cDNA clone SK1-0910 similar to ER glycoprotein, mRNA sequence  
j gi|117828757|gb|EG901453.1|EG901453 EST\_ssal\_evf\_40209 ssalevf mixed\_tissue Salmo salar cDNA Salmo salar cDNA clone ssal\_evf\_553\_261\_fwd 3', mRNA sequence  
k gi|117566103|gb|EG887079.1|EG887079 EST\_ssal\_evf\_45272 ssalevf mixed\_tissue Salmo salar cDNA Salmo salar cDNA clone ssal\_evf\_560\_195\_fwd 3', mRNA sequence

9 SNPs detected

A B C D E F G H I J K L M N O P Q R S T U V W X Y Z a b c d e f g h i j k  cosegregation weighted

368 . G - G - - G - - G - - G G - - - - G G . . . . . . . . . . . . . . . . .   9/9 51.35
369 . G - G - - G - - G - - G G - - - - G G . . . . . . . . . . . . . . . . .   9/9 51.35
370 . T - T - - T - - T - - T T - - - - T T . . . . . . . . . . . . . . . . .   9/9 51.35
371 . A - A - - A - - A - - A A - - - - A A . . . . . . . . . . . . . . . . .   9/9 51.35
372 . G - G - - G - - G - - G G - - - - G G . . . . . . . . . . . . . . . . .   9/9 51.35
373 . T - T - - T - - T - - T T - - - - T T . . . . . . . . . . . . . . . . .   9/9 51.35
374 . T - T - - T - - T - - T T - - - - T T . . . . . . . . . . . . . . . . .   9/9 51.35
375 . G - G - - G - - G - - G G - - - - G G . . . . . . . . . . . . . . . . .   9/9 51.35
376 . G - G - - G - - G - - G G - - - - G G . . . . . . . . . . . . . . . . .   9/9 51.35
